# Cocaine in Urethral Surgery

**Published:** 1893-04

**Authors:** Samuel E. Milliken

**Affiliations:** Lecturer on Surgery in the New York Polyclinic


For Daniel's Texas Medical Journal.

### COCAINE IN URETHRAL SURGERY.

BY SAMUEL E. MILLIKEN, M. D., LECTURER ON SURGERY IN THE NEW YORK POLYCLINIC.

SINCE KOHLLER'S discovery of the local anæsthetic effect of cocaine, which was reported in 1885, the literature on this subject has been voluminous, to say the least. Cocaine has been lauded on the one hand and condemned on the other; nevertheless we must all admit it has reduced minor surgery to comparative simplicity.

That death has resulted from the local use of this drug our medical journals will dèmonstrate, but when it is employed with caution the dangers are slight.

*In Urethral Surgery* some of the most important points to bear in mind are:

FIRST, to get the urinary apparatus in as near a physiological condition as possible before operating, examine the urine daily, endeavor to make it neutral and antispeptic by the use of acid boric, grs. v, three times a day or oil of gaulthia, three drops four times a day.

SECOND. Before examining a patient for stricture the bladder should be respited and the urethra cleansed with some mild antiseptic, such as a $\frac{1}{5000}$ permanganate of potash solution or semisaturate boric acid solution. I prefer the permanganate, particularly when it is necessary to cleanse the bladder, as the various changes in the colour of the solution owing to the amount of deoxidation demonstrate the cleanliness of the tract. After having flushed the bladder three or four times the fluid will pass from the bladder wlthout any appreciable change in colour. The urethra should always be douched from the meatus before the catheter is inserted into the bladder for fear of carrying infection further back, should it already exist.

THIRD. With a properly aseptic tract a small bulbous bogie (24 French) should be used for locating sensative or ulcerated surfaces, prior to the injection of cocaine.

FOURTH. The strength of the cocaine solution should not exceed 6%, while a 2% or 4% will suffice. If a stricture has been located in the anterior part it is well to have the patient make firm pressure in the perineum and thus limit the surface cocainized. One or two drachms of a two or four per cent. solution should be allowed to remain in situ for at least five minutes before the urethratome is inserted.

Locating the stricture may be done by means of the bulbous bougie or Otis' urethrameter; the former is preferable owing to the egg shape, but when more than one stricture exists the urethrameter is more satisfactory owing to one's ability to increase or diminish the size of the bulbs.

Cutting of strictures may be accomplished by the internal or external method; it is usually given in our text books to limit the internal urethrotomy to those within four and one-half or five inches of the meatus; those deeper to be cut by perineal section.

With aseptic precautions a stricture in the membranous portion may be cut instantly with perfect impunity and thus avoid the disagreeable perineal section.

The size of the penis regulates the degree to which the cutting should be carried. Otis gives a rule; e. g. a penis with a circumference of three inches can be cut up to 30 French, and that for each one-fourth inch over three inches, two sizes larger.

A bulbous bougie of the size to which the stricture is cut should be inserted which will locate any constriction that may have escaped the knife. When it is necessary to enlarge the mèatus, which is the case nine times out of ten, it may be accomplished with an ordinary scalpel before the urethratome is inserted. By observing that precaution the whole operation can be performed with little or no pain; otherwise the distention at the meatus produced while the deep stricture is being cut will cause great discomfort.

Immediately after the operation it is very important to douche the urethra with the purmanganate solution, for two reasons: First, asepsis; secondly, to remove any excess of cocaine which may not have escaped previously.

Hemorrhage is a complication which should be guarded against, particularly with deep strictures. Dr. John A. Wyeth's method of applying a figure of eight (8) bandage around the pelvis and under the perineum with a cotton compress over the end of the penis and one over the deep urethra cut is the most satisfactory.

Another important point in detail after cutting the *meatus* is to put in a small pledget of sterilized gauze which does not entirely fill the opening, but simply keeps the recently incised surfaces from coming in contact. If the patient is instructed to reinsert a piece of gauze after each urination, the hemorrhage following the passage of the sound four or five days afterwards will be of little consequence, owing to the rapid healing. It is unnecessary for the compress in the perineum to be worn longer than two or three hours, when the urine may be passed.

*The use of the sound* after the operation is of great importance and must be carried out on principles, only regulated by the type of stricture, as it will require a more frequent use for a broad and firm cicatricial band than for a slight constriction. During the first month, the passage of the sound once a week will usually suffice; afterwards once in two weeks, and so on for three or four months.

It is not advisable to intrust the use of the sound to the patient when it can be avoided, although many good surgeons do so without reluctance. As I have suggested in the beginning of the article, it is essential that the urethra should be cleaned each time before the sound is inserted, particularly where there has been any hemorrhage produced at the previous instrumentation.

Before closing, I wish to state that the worst cases of urethral stricture which have come under my observation, have been those previously treated by dilatation. It seems as though the irritation produced by the distension rather aggravates the formation of adventitious tissue. On the other hand, when the obstruction is removed (by cutting), the methodical use of the sound produces sufficient friction to produce absorption of the cicatricial tissue.

